# The Placentas of Women Who Suffer an Episode of Psychosis during Pregnancy Have Increased Lipid Peroxidation with Evidence of Ferroptosis

**DOI:** 10.3390/biom13010120

**Published:** 2023-01-06

**Authors:** Miguel A. Ortega, Oscar Fraile-Martinez, Cielo García-Montero, Rosa M. Funes Moñux, Sonia Rodriguez-Martín, Coral Bravo, Juan A. De Leon-Luis, Jose V. Saz, Miguel A. Saez, Luis G. Guijarro, Guillermo Lahera, Fernando Mora, Sonia Fernandez-Rojo, Javier Quintero, Jorge Monserrat, Natalio García-Honduvilla, Julia Bujan, Melchor Alvarez-Mon, Miguel Angel Alvarez-Mon

**Affiliations:** 1 Department of Medicine and Medical Specialities, Faculty of Medicine and Health Sciences, University of Alcalá, 28801 Alcalá de Henares, Spain; 2 Ramón y Cajal Institute of Sanitary Research (IRYCIS), 28034 Madrid, Spain; 3 Service of Pediatric, Hospital Universitario Principe de Asturias, 28801 Alcalá de Henares, Spain; 4 Department of Public and Maternal and Child Health, School of Medicine, Complutense University of Madrid, 28040 Madrid, Spain; 5 Department of Obstetrics and Gynecology, University Hospital Gregorio Marañón, 28009 Madrid, Spain; 6 Health Research Institute Gregorio Marañón, 28009 Madrid, Spain; 7 Department of Biomedicine and Biotechnology, Faculty of Medicine and Health Sciences, University of Alcalá, 28801 Alcala de Henares, Spain; 8 Pathological Anatomy Service, Central University Hospital of Defence-UAH Madrid, 28801 Alcala de Henares, Spain; 9 Unit of Biochemistry and Molecular Biology (CIBEREHD), Department of System Biology, University of Alcalá, 28801 Alcalá de Henares, Spain; 10 Psychiatry Service, Center for Biomedical Research in the Mental Health Network, University Hospital Príncipe de Asturias, 28806 Alcalá de Henares, Spain; 11 Department of Psychiatry and Mental Health, Hospital Universitario Infanta Leonor, 28031 Madrid, Spain; 12 Department of Legal Medicine and Psychiatry, Complutense University, 28040 Madrid, Spain; 13 Immune System Diseases-Rheumatology and Internal Medicine Service, University Hospital Príncipe de Asturias, CIBEREHD, 28806 Alcalá de Henares, Spain

**Keywords:** first episode of psychosis, pregnancy, placenta, ferroptosis, lipid peroxidation

## Abstract

Psychosis is a complex entity characterized by psychological, behavioral, and motor alterations resulting in a loss of contact with reality. Although it is not common, pregnancy can be a period in which a first episode of psychosis can manifest, entailing detrimental consequences for both the fetus and the mother. The pathophysiological basis and study of maternofetal wellbeing need to be further elucidated. Lipid peroxidation and ferroptosis are two phenomena that are tightly linked to the placental dysfunction commonly observed in different complications of pregnancy. In the present study, we aim to explore the histopathological and gene expression of different markers of lipid peroxidation and ferroptosis in the placentas of women who underwent a first episode of psychosis during their pregnancy (*n* = 22). The aim is to then compare them with healthy pregnant women (*n* = 20). In order to achieve this goal, iron deposits were studied using Prussian Blue staining. In addition, the protein/gene expression of a transferrin receptor (TFRC), as well as an acyl-CoA synthetase long-chain family member 4 (ACSL-4), arachidonate lipoxygenase-5 (ALOX-5), malondialdehyde (MDA), and glutathione peroxidase 4 (GPX4) were all analyzed through gene expression (RT-qPCR) and immunohistochemical procedures. Our results demonstrate an increased presence of iron deposits that are accompanied by a further expression of TFRC, ACSL-4, ALOX-5, MDA, and GPX4—all of which are observed in the placenta tissue of women who have suffered from a first episode of psychosis. Therefore, in our study, a histopathological increase in lipid peroxidation and ferroptosis markers in the affected women is suggested. However, further studies are needed in order to validate our results and to establish possible consequences for the reported alterations.

## 1. Introduction

New-onset psychosis in pregnancy is a rare but potentially harmful condition for the purposes of maternofetal wellbeing [1]. Psychosis is a phenomenon that is hard to define; it consists of a set of symptoms that result in a loss of contact with reality [2]. The main clinical guidelines, such as the DSM5 (the Diagnostic and Statistical Manual, fifth edition) or ICD-11 (International Classification of Diseases, 11^th^ edition) do not offer a formal definition of psychosis. Nevertheless, they allow one to define psychotic disorders by changes in at least one of the following five domains: delusions; disorganized thought; hallucinations; grossly disorganized or abnormal motor behavior (including catatonia); and negative symptoms [3,4]. In a systematic review and meta-analysis that collected data from 73 articles, Moreno-Küstner et al. [5] found that the prevalence of psychotic disorders was at a rate of 4.6 per 1000 persons, whereas the risk of developing a severe mental illness in pregnancy was around 7.1 in 10,000 women per year [1]. Despite the fact that the estimated incidence of new-onset psychosis in pregnancy has not yet been explored, previous works have established that women who suffered a psychotic episode in pregnancy had an increased risk of multiple adverse obstetric and neonatal outcomes, such as cesarean delivery, poor fetal growth, placental abruption, antepartum/postpartum hemorrhage, fetal abnormalities, as well as distress or stillbirth [6]. In addition, psychosis in pregnancy does indeed represent a huge challenge for clinicians to face, as both medication and maternal illness may have adverse effects on the fetus, thereby demonstrating the need for a balancing between risks, as well as an understanding of the benefits of symptoms and certain therapies [7,8,9].

There are a few studies that have explored the changes in placental tissue within this particular vulnerable group of women. The placenta is a central organ in pregnancy. It exerts many of the physiological functions during pregnancy, (i.e., ensuring an adequate maternofetal exchange, presenting an endocrine activity during pregnancy, and acting as a mechanical, chemical, and immunological barrier). Furthermore, it also determines maternofetal programming, even after gestation [10]. Previous works have defined how there are important structural and functional alterations in the placenta that are derived from suffering different psychiatric conditions during pregnancy [11,12,13,14]. Adequate iron levels and metabolism is essential for the purposes of maternofetal wellbeing in pregnancy. In addition, it has been broadly established that the placenta is a major regulator of iron concentration and metabolism during this period [15]. Notwithstanding the fact that iron deficiency is relatively common in pregnancy, the opposite situation—i.e., iron overload—can also be potentially hazardous during this period. This is due to the fact that labile iron can generate reactive oxygen species, oxidative stress, and lipid peroxidation, which may eventually drive one to a distinctive type of cell death, which is designated as ferroptosis [16]. An increased ferroptosis has been identified in the placental tissue of women who undergo different complications in pregnancy, thereby demonstrating the negative consequences of this process for both the mother and fetus [17,18]. A long list of genetic, morphological, biochemical, and protein markers have been validated in order to study and define ferroptosis in different tissues [19]. In this sense, the aim of the present research consists of the study and analysis of some of the most relevant biomarkers of ferroptosis in the placental tissue of women who have suffered a first episode of psychosis in pregnancy (FE-PW). This is to be achieved in comparison to healthy pregnant women (HC-PW). In choosing this goal, we have included the study of iron deposits (using the histological technique—Prussian blue staining) and—together with the specific proteins involved in this phenomenon—included the transferrin receptor (TFRC); the acyl-CoA synthetase long-chain family member 4 (ACSL-4); arachidonate lipoxygenase-5 (ALOX-5); malondialdehyde (MDA); and glutathione peroxidase 4 (GPX4). The first component, TFRC, is essential for the purposes of cellular uptake and the entry of iron that is bound to ferritin [20]. ACSL-4, ALOX-5, and MDA are major indicators of lipid peroxidation and ferroptosis, whereas GPX4 is considered an important antioxidant that is implicated in the amelioration of both processes [21]. Hence, the gene and protein expression of these components will be performed by using RT-qPCR and immunohistochemistry, respectively.

## 2. Patients and Methods

### 2.1. Study Design

An observational, analytical, and prospective study was conducted regarding 42 women in the third trimester of pregnancy (FE-PW = 22; HC-PW= 20). The median age in the FE-PW group was 33.5 (21–42) years and the median gestational age was 40 (38–41) weeks. For HC-PW, the median age was 33.5 (25–39) years and the median gestational age was 40 (39–42) weeks. The sociodemographic and clinical features of these patients are summarized in Table 1.

A psychiatrist confirmed the diagnosis of FE-PW according to the DSM-5 criteria, using the structured clinical interview for DSM-5 (SCID-5) [22]. Then, the symptoms were assessed using the positive and negative syndrome scale (PANSS) [23]. Among the inclusion criteria there were: (1) Pregnant women aged between 18 to 45 years and (2) fluent Spanish-speaking women, who thus enabled a proper assessment to be conducted. On the other hand, the exclusion criteria were: (1) Meeting the diagnostic criteria for another current Axis-I mental disorder or for intellectual disability and (2) a prior history of neurodevelopmental disorders or head injuries in conjunction with a loss of consciousness. Each subject was properly informed prior to enrolment. In addition, all of them provided their signed written consent. This work was approved by the Clinical Research Ethics Committee of the Central University Hospital of Defense University of Alcalá (37/17). Furthermore, the study was conducted in agreement with the ethical principles of autonomy, beneficence, non-maleficence, distributive justice, the regulations of Good Clinical Practice, as well as the principles detailed in the Declaration of Helsinki (2013) and the Oviedo Convention (1997).

### 2.2. Sample Collection and Processing

Placental biopsies were collected immediately after delivery from both the FE-PW and HC-PW groups. Five placental pieces were obtained for every sample and by the use of a scalpel. Moreover, mixed cotyledons were included and divided into two different sterile tubes: one with an RNAlater^®^ solution (Ambion; Thermo Fisher Scientific, Inc., Waltham, MA, USA) and another containing a minimal essential medium (MEM; Thermo Fisher Scientific, Inc., Waltham, MA, USA) with 1% of antibiotic/antimycotic (streptomycin, amphotericin B, and penicillin; Thermo Fisher Scientific, Inc.). Afterward, placental samples were processed within a sterile environment in a class II laminar flow hood (Telstar AV 30/70 Müller 220 V 50 MHz; Telstar; Azbil Corporation, Tokyo, Japan). Subsequently, samples were conserved in 1 mL of RNAlater^®^ (−80 °C) for the purposes of later processing and gene expression study. Preserved MEM samples were washed and rehydrated 5 times in an MEM that was free of antibiotics in order to remove erythrocytes. Each sample was then cut by using another scalpel into 2 cm fragments, later being fixed in F13 (i.e., 60% ethanol, 20% methanol, 7% polyethylene glycol, and 13% distilled water) according to well-established protocols [24]. Thereafter, paraffin-embedded samples were developed using specific molds and once the paraffin was solidified, an HM 350 S rotary microtome (Thermo Fisher Scientific, Inc., Waltham, MA, USA) was used to cut 5 µm thick sections. After this, the samples were then collected after being placed in a hot water bath onto glass slides that were treated with 10% polylysine. Eventually, these samples were used to perform the histological and immunohistochemical techniques.

### 2.3. Histological and Immunohistochemical Techniques

Initially, deparaffination of the samples was fulfilled by using xylol, decreasing concentrations of ethanol (i.e., 100%, 96%, and 70%), and then, finally, they were hydrated in distilled water. From this point, we conduct two main types of histological techniques: Perls’/Prussian Blue staining and immunohistochemistry.

Prussian Blue staining was performed by using the Kit HistoPerls (REF. 361850-0000 RAL Diagnostics - Site Montesquieu - Martillac – France), following the instructions given by the manufacturer: (1) After sample hydration, the samples were stained with potassium ferrocyanide solution for 30 min; (2) rinsed with distilled water; (3) staining was conducted with a nuclear red for 10 min; (4) then rinsed with distilled water again; and then (5) dehydrated with growing concentrations of ethanol and finally with xylol. Eventually the cover slides were mounted in a xylol-based medium (Canada balsam). With this procedure, ferric ions react with potassium ferrocyanide in an acid environment, thereby leading to the precipitation of ferric ferrocyanide (or Prussian blue)—thus allowing the detection of hemosiderin.

Immunohistochemical studies were completed through an avidin–biotin complex with avidin peroxidase, thereby facilitating antigen/antibody reaction detection. This was achieved by following the process as described in previous protocols [25]. Placental tissues were washed three times with PBS 1× for 5 min. Then, non-specific binding sites were blocked after 30 min with a 3% bovine serum albumin (BSA), which was diluted in PBS at room temperature (RT). Afterward, samples were incubated with the primary antibody for 90 min and, subsequently, with a 3% BSA Blocker (cat. no. 37,525; Thermo Fisher Scientific, Inc., Waltham, MA, USA) and PBS (at 4 °C, overnight). The following day, placental samples were incubated with biotin-conjugated secondary antibodies, which were previously diluted in PBS for 90 min at RT (Table 2). Then, the avidin–peroxidase conjugate ExtrAvidin^®^-Peroxidase (Sigma-Aldrich; Merck KGaA, San Luis, MO, USA) was added for one hour at room temperature (1:200 dilution in PBS) and, eventually, a chromogenic diaminobenzidine (DAB) substrate kit (cat. no. SK-4100; Maravai LifeSciences, CA, USA) was used. Further, it was prepared immediately before exposure (5 mL distilled water; four drops DAB; two drops of hydrogen peroxide; and two drops of buffer) and was used in order to determine protein expression level.

The use of the peroxidase chromogenic substrate for 15 min at RT allowed the detection of the signal in the form of a brown staining. Negative controls (i.e., replacing incubation with a primary antibody for a PBS solution) were assigned for each protein and Carazzi hematoxylin was used for 15 min in order to determine the contrast in all tissues. The antibodies employed in our study are detailed in the protocol specifications (Table 2).

### 2.4. Lipid Peroxidation Assay

A colorimetric lipid peroxidation assay kit (ab118970; Abcam, Cambridge, UK) was employed for the sensitive detection of MDA in the placental tissue of both FE-PW and HC-PW. This procedure enables one to detect MDA, as this component reacts with the present thiobarbituric acid (TBA) in order to generate an MDA-TBA adduct that is quantified colorimetrically (OD 532 nm). The first part of the experiment was conducted using established protocols that were previously described by Ortega et al. [26]. Then, for the histological study, we used 20 mg of placental tissue and processed it according to the following protocol: (1) Rinse the tissue in cold PBS; (2) prepare the lysis solution at 300 mL of lysis buffer with MDA and 3 µL of BHT (100×); (3) homogenize the tissue in 303 µL of a lysis solution (buffer + BHT) with 10–15 passes with a homogenizer on ice; and then (4) centrifuge at 13,000 × *g* for 10 min in order to remove the insoluble material and to thus collect the supernatant.

### 2.5. Gene Expression Study

The guanidinium thiocyanate-phenol-chloroform method was performed in order to allow the RNA extraction as described in previous works [27]. This method facilitates the analysis of the mRNA expression levels of selected genes.

In addition, complementary DNA (cDNA) was synthesized by a reverse transcription (RT) from 50 ng/µL of the RNA samples. A total of 4 µL of each sample was mixed with 4 µL of a 0.25 µg/µL oligo-dT solution (Thermo Fisher Scientific, Inc., Waltham, MA, USA). Then, it was placed at 65 °C for 10 min in a dry bath (AccuBlock, Labnet International Inc., New Jersey, USA), thereby facilitating RNA denaturation. Then, samples were put on ice with 10 µL of the RT mix containing the following products: 2.8 µL of First Strand Buffer 5X, composed by 250 mM of Tris-HCl and a pH of 8.3; 375 mM of KCl:15 mM MgCl2 (Thermo Fisher Scientific, Inc., Waltham, MA, USA); 1 µL of retrotranscriptase enzyme (all from Thermo Fisher Scientific, Inc., Waltham, MA, USA); 2 µL of 10 mM deoxyribonucleotide triphosphate; 2 µL of 0.1 M dithiothreitol; 1.7 µL of DNase and RNase free water; and 0.5 µL of RNase inhibitor (RNase Out).

A G-Storm GS1 thermocycler (G-Storm Ltd., Somerset, UK) was used to maintain RT. The samples were incubated at 37 °C for 1 h and 15 min in order to enhance cDNA synthesis. Then, the temperature was raised up to 70 °C and held for 15 min, thereby causing retrotranscriptase denaturation. Thereafter, the temperature was gradually reduced to 4 °C. Negative RT was equally performed in order to ensure the lack of genomic DNA contamination in RNA samples, in which the M-MLV reverse transcriptase was exchanged by DNase- and RNase-free water. Moreover, the cDNA produced at room temperature was diluted in DNase- and RNase-free water (1:20) and kept at −20 °C until further use was required.

In Table 3, specific primers for each gene are summarized. The design was performed through the Primer-BLAST and AutoDimer online applications [28,29]. We used the constitutively expressed gene, the TATA-box binding protein (TBP), as a control in order to normalize our results [30]. Gene expression units are expressed as relative quantities of mRNA. Further, RT-qPCR was performed on a StepOnePlus™ System (Applied Biosystems; Thermo Fisher Scientific, Inc.) using the relative standard curve method. The reaction occurred as follows: 5 µL of the sample mixed at 1:20 with 10 µL of a iQ™ SYBR^®^ Green Supermix (Bio-Rad Laboratories, Inc., Hercules, California, USA); then, it was mixed with 1 µL each of the forward and reverse primers; and, next, 3 µL of the DNase and RNase-free water was mixed in—which were then added to a MicroAmp^®^ 96-well plate (Applied Biosystems; Thermo Fisher Scientific, Inc., Waltham, MA, USA). The thermocycling conditions were: (1) An initial denaturation for 10 min at 95 °C; (2) a denaturation for 15 s at 95 °C; (3) an annealing at variable temperatures according to the melting temperature of each primer pair for 30 s; and an elongation at 72 °C for 1 min, for 40–45 cycles. Then, a dissociation curve for 15 s at 95 °C, 1 min at 60 °C, 15 s at 95 °C, and 15 s at 60 °C was developed. Fluorescence detection was performed at the end of each repeat cycle (amplification) and at the different steps of the dissociation curve. Data collected from the genes were included in a standard curve made by serial dilutions of a mixture of samples, which were then included in each plate according to the constitutive expression of TBP (in agreement with the manufacturer’s protocols). In order to obtain consistent results, this RT-qPCR was performed twice in all samples of placenta tissue.

### 2.6. Histological and Statistical Analysis

For each histological sample, five different sections and ten fields were, at random, examined by two independent histologists. Immunohistochemical expression was classified as positive when the stained mean area in the studied sample was ≥5%, thereby following the immunoreactive score (IRS) as established in previous studies [31]. This method allows one to classify immunostaining as per the following scales—0–1: minimum staining (≤25%); 2: moderate staining (25–65%); and 3–4: strong staining (≥65–100%). A Zeiss Axiophot optical microscope (Carl Zeiss, Oberkochen, Germany) was used for the purposes of the histopathological determinations. Statistical analyses were conducted with a GraphPad Prism^®^ v6.0 (GraphPad, Inc., San Diego, CA, USA) program and a Mann–Whitney U test was performed in order to compare between both groups. Results were expressed as the median ± SD. Significant values were established as *p* < 0.05 (*), *p* < 0.01 (**), and *p* < 0.001 (***).

## 3. Results

### 3.1. The Placental Tissue of Women with a First Episode of Psychosis in Pregnancy Show Evidence of Further Iron Deposits Together with an Enhanced Expression of the Transferrin Receptor Gene and Protein

**Figure 1:**
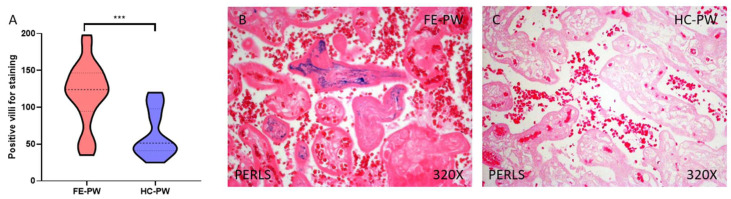
Histopathological study of iron deposits using the Perls’/Prussian Blue staining for positive villi (**A**), in the FE-PW (**B**), and HC-PW (**C**) group. FE-PW = first psychotic episode during pregnancy. HC-PW = healthy controls pregnant women. *p* < 0.001 (***).

First of all, we evaluated—via Prussian Blue staining—the presence of ferric ions that were ligated to hemosiderin. Our results indicated that the placental tissue of FE-PW women show increased iron deposits in comparison to HC-PW (FE-PW = 118.545 ± 43.541; HC-PW = 63.650 ± 31.982; *** *p* < 0.001, Figure 1A). Histologically, we appreciate that the increased iron staining affected the totality of the villous structure, including syncytiotrophoblasts, cytotrophoblasts, the matrix, and fetal capillaries (Figure 1B).

**Figure 2:**
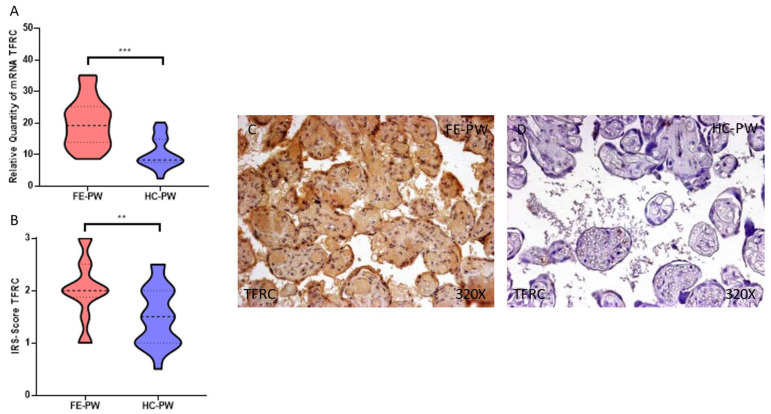
(**A**) TFRC mRNA expression in both the FE-PW (first episode of psychosis in pregnancy) and HC-PW groups (healthy controls). (**B**) IRS-Scores for TFRC expression in the placenta villi of the FE-PW group and the HC group. (**C**,**D**) Images showing the immunostaining for TFRC in the placenta villi of the FE-PW group and the HC-PW group. *p* < 0.01 (**), *p* < 0.001 (***).

On the other hand, we evaluated the gene and protein expression of TFRC in the placentas of both FE-PW and HC-PW through RT-qPCR and immunohistochemistry, respectively. Our results help to define the fact that FE-PW display a significant increase in the gene expression of TFRC (FE-PW = 19.983 ± 7.635 relative quantity mRNA [RQ]; HC-PW = 10.388 ± 4.801 RQ; *** *p* < 0.001, Figure 2A). In respect to protein expression, we observed that TFRC expression was significantly higher expressed in FE-PW (FE-PW = 2.023 ± 0.587; HC-PW = 1.500 ± 0.562, ** *p* = 0.006, Figure 2B). Following histopathological observations, TFRC appears to be broadly expressed in the different cells of the villous structure (Figure 2C).

### 3.2. The Placentas of Women with a First Episode of Psychosis in Pregnancy Display a Significant Augmentation of Lipid Peroxidation and Ferroptosis Markers

Secondly, we investigated the gene and protein expression of different markers regarding lipid peroxidation and ferroptosis, including ACSL4 and ALOX5 by RT-qPCR, as well as the immunohistochemical studies in the placentas of women with FE-PW, which were then compared with HC-PW.

**Figure 3:**
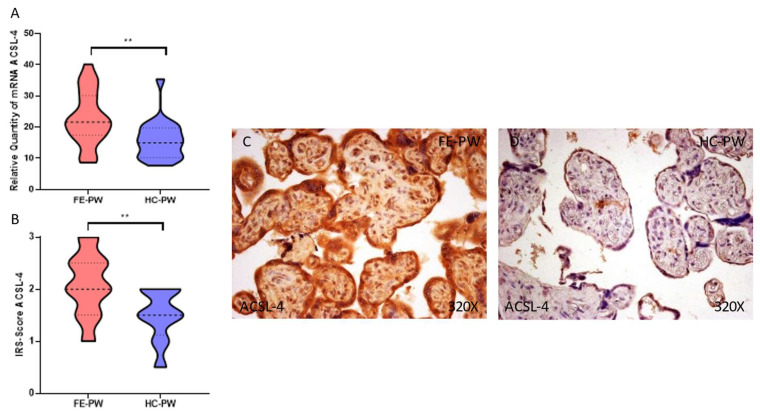
(**A**) ACSL-4 mRNA expression in both the FE-PW (first episode of psychosis in pregnancy) and HC-PW groups (healthy controls). (**B**) IRS-Scores for ACSL-4 expression in the placenta villi of the FE-PW group and the HC group. (**C**,**D**) Images showing the immunostaining for ACSL-4 in the placenta villi of the FE-PW group and the HC-PW group. *p* < 0.01 (**).

Regarding ACSL-4, we report a significant upregulation of the gene expression of this component in FE-PW (FE-PW = 22.797 ± 8.649 RQ; HC-PW = 15.803 ± 6.268 RQ, ** *p* = 0.002, Figure 3A). Regarding protein expression, we observed a significant increase in ACSL-4 expression within the placental tissue of FE-PW when compared to HC-PW (FE-PW = 2.091 ± 0.590; HC-PW = 1.500 ± 0.487, ** *p* = 0.0016, Figure 3B). In addition, the histopathological expression of ACSL-4 can be observed in the totality of the placental villi of FE-PW (Figure 3C).

**Figure 4:**
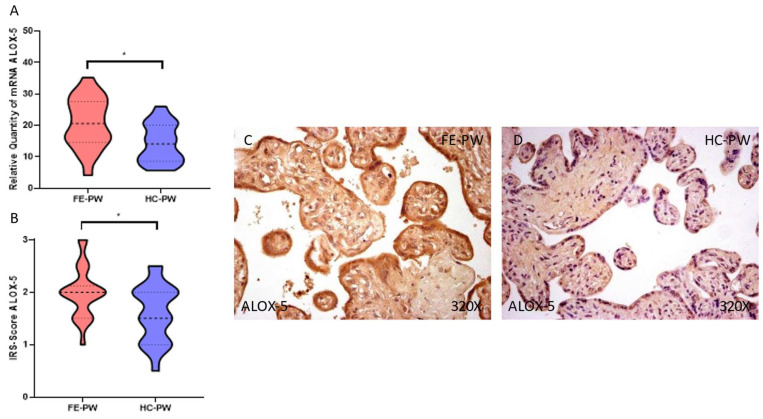
(**A**) ALOX-5 mRNA expression in both the FE-PW (first episode of psychosis in pregnancy) and HC-PW groups (healthy controls). (**B**) IRS-Scores for ALOX-5 expression in the placenta villi of the FE-PW group and the HC group. (**C**,**D**) Images showing the immunostaining for ALOX-5 in the placenta villi of the FE-PW group and the HC-PW group. *p* < 0.05 (*).

Simultaneously, the gene expression of ALOX-5 was also significantly higher in FE-PW (FE-PW = 20.678 ± 8.046 RQ versus HC-PW = 14.293 ± 6.157 RQ; * *p* = 0.0111 Figure 4A). Regarding protein expression, we report a significant increase in ALOX-5 in the FE-PW group (FE-PW = 2.000 ± 0.488; HC-PW = 1.600 ± 0.553, * *p* = 0.0293, Figure 4B). When observed with a microscope, the augmented ALOX-5 expression was found in the different parts of the villi, including syncytiotrophoblasts, cytotrophoblasts, the matrix, and fetal capillaries (Figure 4C).

**Figure 5:**
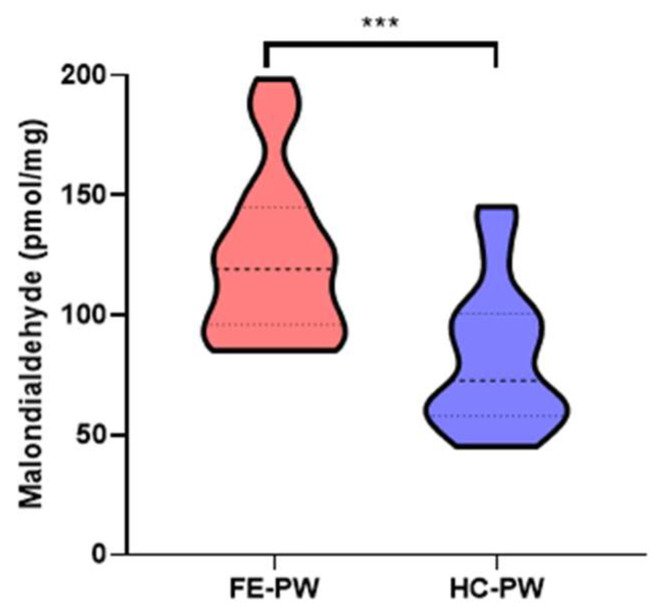
Malondialdehyde (MDA) levels, in pmol/mg, found in the placental tissues of both the FE-PW and HC-PW groups. FE-PW = first psychotic episode during pregnancy. HC-PW = healthy controls pregnant women. *p* < 0.001 (***).

Finally, the levels of MDA in the placentas of the FE-PW group were significantly higher than those found in the HC group (FE-PW = 122.965 ± 34.259 pmol/mg; HC-PW = 81.821 ± 30.026 pmol/mg, *** *p* < 0.001, Figure 5).

### 3.3. Placental Tissue of Women with a First Episode of Psychosis during Pregnancy Present a Gene and Protein Overexpression of Glutathione Peroxidase 4

**Figure 6:**
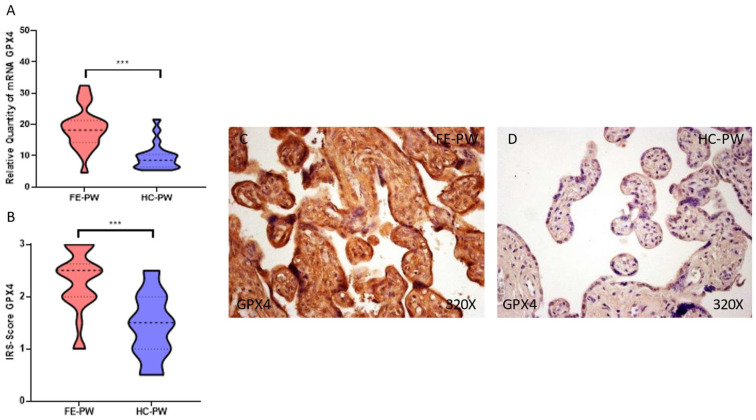
(**A**). GPX-4 mRNA expression in both the FE-PW (first episode of psychosis in pregnancy) and HC-PW groups (healthy controls). (**B**). IRS-Scores for GPX-4 expression in the placenta villi of the FE-PW group and the HC group. (**C**,**D**). Images showing the immunostaining for GPX-4 in the placenta villi of the FE-PW group and the HC-PW group. *p* < 0.001 (***).

Finally, we analyze the gene and protein expression of the antioxidant GPX4 in the FE-PW and HC-PW groups. In terms of gene expression, we observed that GPX4 was significantly higher in the FE-PW group (FE-PW = 18.843 ± 6.587 RQ; HC-PW = 9.752 ± 4.208 RQ, *** *p* < 0.001, Figure 6A). Moreover, the immunohistochemical detection of GPX4 was also significantly higher in the FE-PW (FE-PW = 2.295 ± 0.591; HC-PW = 1.425 ± 0.613, *** *p* < 0.001, Figure 6B). Indeed, it was detected throughout the entire villous structure (Figure 6C).

## 4. Discussion

Our results demonstrate that the placental tissue of women who had undergone a first episode of psychosis during pregnancy display an enhanced iron accumulation, lipid peroxidation, as well as increased ferroptosis markers when compared to the placentas studied in the HC group. These alterations can contribute to and partially explain the detrimental consequences of psychosis in pregnancy, thereby aiding one to understand the negative implications for the fetus and the mother.

Ferroptosis is an iron-dependent form of programmed cell death (PCD) resulting from the accumulation of lipid-reactive oxygen species, lipid peroxidation, and the depletion of plasma membrane polyunsaturated fatty acids (PUFAs) [32]. This process is influenced by multiple cellular events; including redox homeostasis; iron handling; hypoxia; mitochondrial activity or the metabolism of carbohydrates; as well as lipids and amino acids, among other factors [33]. Previous studies have demonstrated that ferroptosis may play a pivotal role in the placental dysfunction that is observed in different pregnancy complications, such as oxidative stress, lipid peroxidation, and hypoxia-reoxygenation injury—which are all common phenomena that are tightly linked to these conditions [17].

There are many markers that may be indicators for an increased ferroptosis in different tissues. In our study, we have demonstrated an altered expression of critical components that are potentially associated with ferroptosis. This is supported by the presence of TFRC, ACSL-4, ALOX-5, GPX-4, and MDA. In addition, an exacerbated iron accumulation is differentially observed in the placental tissue of FE-PW when compared to HC-PW.

Through the process of Prussian blue staining, we show that there is an increased presence of iron in the placental tissue of FE-PW. By using this technique, previous studies have demonstrated that the placental tissue possessed an enhanced iron accumulation under different obstetric diseases [34,35]. This fact, together with the higher expression of the iron transporter TFRC, can understood as indicative of an enhanced iron accumulation and entry in the trophoblasts of women who are affected by a first episode of psychosis. TFRC is highly expressed on the apical maternal-facing membrane of the syncytiotrophoblasts (STBs) [36], which are the multinucleated, specialized layer of epithelial cells that are in contact with the maternal blood—which also orchestrate the maternofetal exchange [10]. The function of TFRC is to ensure a proper transfer of iron from the maternal to fetal blood. Mechanistically, iron uptake from transferrin involves the binding of transferrin to the TFRC, as well as an internalization of transferrin within an endocytic vesicle via receptor-mediated endocytosis and the release of iron from protein by a decrease in endosomal pH [37]. Prior works have evidenced an aberrant expression of TFRC in different obstetric complications, such as intrauterine growth restriction, preeclampsia, or gestational diabetes [38,39,40]. Beyond these, the increased expression of TFRC has been validated as a feasible marker of ferroptosis in multiple cell cultures and tissues [41]. Thus, the enhanced expression of TFRC in FE-PW may likely be an indicator of ferroptotic cell death in the placenta of these women, thereby increasing the intracellular levels of iron in the cell. After the excessive entry of iron through the TFRC, different free radicals can be formed, especially reactive oxygen species (ROS) or reactive nitrogen species (RNS). This process is known as the Fenton reaction, whereby both hydroxyl radicals and higher oxidation states of the iron are generated [42]. Concomitantly, the proper activity regarding the mitochondria together with specific plasma membrane enzymes—such as NADPH oxidases (NOX)—can equally generate an enhanced production of free radicals, which is required for the process of lipid peroxidation and ferroptosis [43].

However, not only is the increased production of ROS and RNS required, but also it is noted that other critical pathways need to be activated in order to promote or facilitate an increase in lipid peroxidation and ferroptosis. ACSL4 is an enzyme that is implicated in the enrichment of cellular membranes with long ω6 PUFA. Moreover, it is considered a central component for the purpose of ferroptosis execution [44]. Hence, the main function of ACSL4 is to increase the content of PUFAs in the cell, which are more prone to suffer the oxidative reactions that are required in order for ferroptosis to occur. Recently it has been shown that the SARS-CoV-2 infection can induce an increased expression of ACSL4 in the placenta of pregnant women, thereby enhancing the process of ferroptosis in this tissue [45]. Despite the association between ACSL4 with ferroptosis, previous works have also evidenced the fact that animals with ACSL4 depletion can also undergo ferroptosis under certain conditions [21]. This, therefore, demonstrates that the single expression of this component is not enough to define ferroptosis, but it can aid one in understanding the increased susceptibility of the affected tissues once one undergoes this process. Furthermore, ALOX-5 is another enzyme that was upregulated in our study. ALOX-5 is an iron-containing and nonheme dioxygenase that catalyzes the peroxidation of PUFAs, such as arachidonic acid [46]. This enzyme is implicated in the biosynthesis of leukotrienes, the modulation of the inflammatory responses, as well as different types of cell death—such as apoptosis, pyroptosis, and the proper ferroptosis [47]. Previous works have showed that the placental tissue of women with preeclampsia display an enhanced expression of ALOX-5, as well as the fact that they are involved in lipoxin A4 (LXA4) deficiency [48]. Interestingly, an exposure to glucocorticoids appears to be directly related to the increased expression of ALOX-5 in the placenta [49]. In this sense, a meta-analysis conducted by Hubbard and Miller [50] demonstrated how patients with a first episode of psychosis present abnormally elevated levels of circulating glucocorticoids (cortisol). Therefore, high circulating levels of glucocorticoids in FE-PW can be understood to be directly involved in ALOX-5 overexpression, as well as the enhanced ferroptosis cell death that is observed in the placenta tissue of these women. Lastly, MDA appears to be equally upregulated in our study. MDA is one of the final products of lipid peroxidation. It exerts a high mutagenic capacity and is an indicator of oxidative stress in different cells and tissues [51,52]. Previous works have evidenced an upregulated MDA detection in the maternal and fetal structures of women under pathological pregnancies [26,53]. Thus, the increased detection of MDA corroborates the increased lipid peroxidation that is observed in FE-PW.

**Figure 7:**
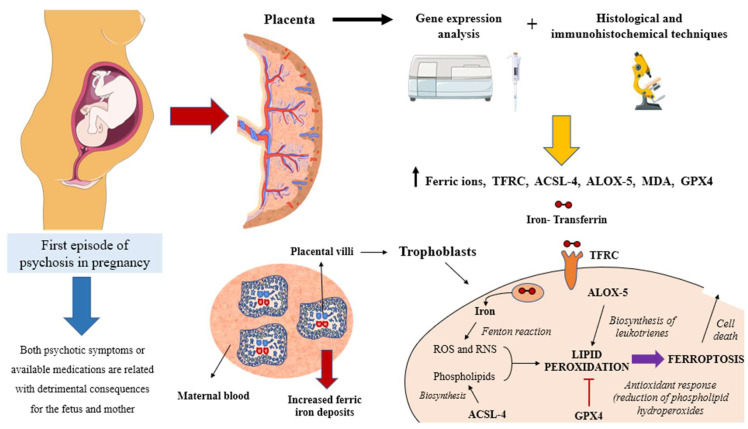
A global view of the main aspects and implications discussed in this work. As showed prior, a first episode of psychosis in pregnancy entails multiple challenges for healthcare professionals. This is because both the symptoms or possible interventions may have detrimental consequences for both the fetus and the mother. In the present work, we have demonstrated that the placentas of these women present an increased gene and protein expression of different components that are directly involved in the regulation of ferroptosis and lipid peroxidation. This fact, together with an increased detection of ferric ions, may be an indicator of iron accumulation in the placenta, thereby defining a possible pathophysiological role from these disturbances.

Finally, we observe an overexpression of GPX4 in the placenta tissue of women who have suffered a first episode of psychosis. GPX4 is one of the main antioxidant mediators in the organism. Mechanistically, GPX4 is a selenoenzyme involved in the reduction in phospholipid hydroperoxides (PLOOH). This is achieved by using reduced glutathione (GSH) [54]. In more detail, PLOOH is both a product and the main initiator of peroxidative chain reactions, whereas GPX4, GSH, and α-tocopherol are integrated in a concerted antiperoxidative mechanism [55]. In addition, GPX4 is considered a major regulator of the ferroptosis process [55,56]. This enzyme is required for ameliorating the lipid peroxidation within the cell as if a threshold is exceeded, which can also mean that ferroptosis can occur [57]. GPX4 activity is required for the protection of trophoblasts against ferroptosis [17]. However, there are two major mechanisms that are recognized as key for ferroptosis: the extrinsic and the intrinsic pathway. The intrinsic pathway is mainly induced by reducing the activity or expression of different antioxidants, such as GPX4; whereas, the extrinsic pathway is initiated through the regulation of different transporters, including the inhibition of the amino acid or through an increase in iron transporters, such as TFRC [43]. As summarized in Figure 7, the notable increase in TRFC, together with other markers (i.e., ACSL4, ALOX-5, and MDA) indicates that an enhanced lipid peroxidation and ferroptosis is observed in the placenta tissue of FE-PW. Furthermore, the antioxidant GPX4 is upregulated in order to limit the extent of peroxidative damage and ferroptotic cell death that is observed in this organ.

Overall, our study is in accordance with previous works that have been conducted. In addition, we have also evidenced an association between changes in placental morphology and function with mental disorders in pregnancy [12]. This relationship seems to be bidirectional, as the placenta may influence the onset of psychosis and, in turn, become modulated by mental disorders in pregnancy (which may result in an echo in the offspring [58]). In this sense, the results of our study suggest that the increased lipid peroxidation and ferroptosis observed in the placental tissue of FE-PW are somehow related to a first episode of psychosis in pregnancy. However, it is difficult to study if these changes can contribute to its development or if it is a consequence of having suffered it. As we have studied the placentas of women after delivery, we could only confirm that there is an association between having undergone a first episode of psychosis in pregnancy and an increased ferroptosis and lipid peroxidation in the placenta. Furthermore, such a phenomenon was equally observed in different obstetric conditions. Further studies are required in order to better understand the role and implications of the placental changes in the development or consequences of psychosis during pregnancy.

## 5. Conclusions

In this study, we have found an increased detection of extracellular iron in the placental tissue of FE-PW, together with an intracellular augmentation of TFRC, ACSL4, ALOX-5, MDA, and GPX4. These results suggest that suffering from a new-onset psychotic episode in pregnancy may promote lipid peroxidation and ferroptotic cell death in maternofetal structures, such as the placenta. Future studies are warranted in order to corroborate or deepen on the observed histopathological alterations and possible consequences for the maternofetal wellbeing in these patients.

## Figures and Tables

**Table 1:** Clinical and demographic characteristics of women with a first-psychotic episode during pregnancy and in the healthy controls. FE-PW = first-psychotic episode during pregnancy. HC-PW = healthy controls pregnant women.

	FE-PW (*n* = 22)	HC-PW (*n* = 20)
Median age (IQR), years	33.5 (21–42)	33.5 (25–39)
Median gestational age (IQR), weeks	40 (38–41)	40 (39–42)
C-section delivery, *n* (%)	3 (13.6)	2 (10.0)
Previous pregnancies, *n* (%)	8 (36.4)	9 (45.0)
Previous abortions, *n* (%)	1 (4.5)	2 (10.0)
Regular menstrual cycles, *n* (%)	17 (77.3)	16 (80.0)
PANSS mean (SD)	Positive 18.8 (6.3)	-----
	Negative 25.7 (7.9)	

**Table 2:** Primary and secondary antibodies used and their dilutions.

Antigen	Species	Dilution	Provider	Protocol Specifications
TFRC	Rabbit Monoclonal	1:500	Abcam (ab185550)	EDTA pH = 9, before incubation with blocking solution
ACSL-4	Rabbit Monoclonal	1:100	Abcam (ab155282)	100% Triton, 0.1% in PBS for 10 min, before incubation with blocking solution
ALOX-5	Rabbit Monoclonal	1:250	Abcam (ab169755)	100% Triton 0.1% in PBS for 10 min, before incubation with blocking solution
GPX4	Rabbit Monoclonal	1:100	Abcam (ab125066)	10 mM Sodium citrate pH = 6, before incubation with blocking solution
IgG (Rabbit)	Mouse	1:1000	Sigma-Aldrich (RG-96/B5283)	------

**Table 3:** Primers used for RT-qPCR: sequences and binding temperatures (Temp).

GENE	SEQUENCE Fwd (5′→3′)	SEQUENCE Rev (5′→3′)	Temp
TBP	TGCACAGGAGCCAAGAGTGAA	CACATCACAGCTCCCCACCA	60 °C
TFRC	GAACTACACCGACCCTCGTG	GTGCTGTCCAGTTTCTCCGA	60 °C
ACSL-4	GCTGGGACAGTTACTGAAGGT	AGAGATACATACTCTCCTGCTTGT	58 °C
ALOX-5	TGGCGCGGTGGATTCATAC	AGGGGTCTGTTTTGTTGGCA	60 °C
GPX4	ATTGGTCGGCTGGACGAG	CCGAACTGGTTACACGGGAA	59 °C

## Data Availability

The data used to support the findings of the present study are available from the corresponding author upon request.
